# Real-Time Temperature Rise Estimation during Irreversible Electroporation Treatment through State-Space Modeling

**DOI:** 10.3390/bioengineering9100499

**Published:** 2022-09-23

**Authors:** Sabrina N. Campelo, Edward J. Jacobs, Kenneth N. Aycock, Rafael V. Davalos

**Affiliations:** Virginia Tech-Wake Forest School of Biomedical Engineering and Sciences, Virginia Tech Department of Biomedical Engineering and Mechanics, Virginia Tech, Blacksburg, VA 24061, USA

**Keywords:** pulsed field ablation, PFA, electroporation, H-FIRE, thermal mitigation, temperature prediction, black-box modeling, agar phantom

## Abstract

To evaluate the feasibility of real-time temperature monitoring during an electroporation-based therapy procedure, a data-driven state-space model was developed. Agar phantoms mimicking low conductivity (LC) and high conductivity (HC) tissues were tested under the influences of high (HV) and low (LV) applied voltages. Real-time changes in impedance, measured by Fourier Analysis SpecTroscopy (FAST) along with the known tissue conductivity and applied voltages, were used to train the model. A theoretical finite element model was used for external validation of the model, producing model fits of 95.8, 88.4, 90.7, and 93.7% at 4 mm and 93.2, 58.9, 90.0, and 90.1% at 10 mm for the HV-HC, LV-LC, HV-LC, and LV-HC groups, respectively. The proposed model suggests that real-time temperature monitoring may be achieved with good accuracy through the use of real-time impedance monitoring.

## 1. Introduction

Irreversible electroporation (IRE) and second-generation high-frequency irreversible electroporation (H-FIRE) are techniques currently being developed to treat malignant tumors when other treatment methods, such as surgical resection or thermal ablation, are not appropriate [1]. It is also being developed for the treatment of arrhythmogenic cardiac tissues [2,3]. This technique uses short (1–100 μs) high-magnitude electric pulses (1–3 kV) to produce an electric field resulting in an increase in transmembrane potential (TMP), leading to cell death within the target zone [4,5,6]. When this TMP limit is reached (∼1 V), nanoscale defects, or “pores”, form in the cellular membrane. When numerous pulses are administered, the formation of large, long-lived pores disrupts cellular homeostasis, resulting in cell death via different mechanisms [7,8]. Because cell death with IRE is primarily dependent on the generated TMP, IRE is classified as a non-thermal ablation method and may thus be used in a variety of settings. An electric field is produced between two or more needle electrodes placed directly into the tumor-containing area prior to pulse administration. In some cases, a single-insertion bipolar probe (two electrodes integrated into the same cylindrical shaft) is employed.

It has been demonstrated with IRE that ablation and thermal effects can be mutually exclusive phenomena [9,10,11]. Innumerable follow-up studies have found appropriate parameters for ablation in various contexts. However, unfavorable side effects caused by temperature increases continue to be the most important issue limiting the size of the IRE ablations [12]. Clinically, discrepancies in pulse delivery methods and clinical expertise might result in variable degrees of thermal tissue damage, which is most likely to blame for the wide range of complication rates and oncological outcomes documented in the literature [13,14,15,16]. Even at low voltages, the steep potential gradient at the electrode borders can generate quite large electric fields (and consequently temperatures), causing tissue coagulation, extracellular protein denaturation, and bleeding [17,18]. This may compromise the safety of therapy if the electrodes are placed in close proximity to or in direct contact with specific anatomical structures sensitive to thermal damage — for instance, in the liver, thermal injury to the bile ducts can lead to life-threatening consequences [17,19].

Despite these reservations, present clinical pulsing methods do not actively take temperature increases into account during IRE treatments. In the absence of real-time feedback during pulse delivery, the risk of thermal injury varies greatly across patients depending on the tissue being treated, electrode design, pulsing paradigm, and patient-specific tissue parameters. A recent study, for example, suggested that around 30% of the typical IRE ablation volume suffers from moderate hyperthermia (40–50 ${}^{{}^{\circ}}$C), with 5% exposed to temperatures over 50 ${}^{{}^{\circ}}$C [20]. Maintaining the nonthermal elements of IRE will become more challenging as clinicians and researchers strive to increase ablation volumes in order to treat bigger tumors. Other factors to consider include that IRE performed in proximity to a metal stent has demonstrated higher temperatures surrounding the electrodes and viable tissue remnants [21]. Furthermore, current research shows that, even when thermal damage is improbable, lowering mild-to-moderate thermal impacts may encourage greater immune activation, which may improve long-term therapeutic effectiveness and patient outcomes [22,23,24]. As a result, strategies to reduce and/or manage thermal effects in the proximity of the electrodes are critical to improving IRE and other pulsed electric field (PEF)-based ablation modalities for wider clinical usage.

Several thermal mitigation (TM) solutions have been proposed to decrease the temperature increase during PEF therapy. Innovative probe designs that include heat-dissipating technology such as phase-change materials or active cooling [25,26], as well as pulse paradigm changes that enable tissue perfusion to distribute heat between successive pulses [27,28], have demonstrated an ability to reduce thermal damage. However, the advantages of these techniques must be weighed against the increased time, cost, and effort required by modified probes [20,29,30]. While previous studies have utilized computational modeling for prospective and retrospective estimations of temperature rises during treatment, no studies have been conducted to implement real-time temperature monitoring without requiring the insertion of additional sensor devices.

In this study, we develop a mathematical model to estimate real-time tissue temperature changes at distances 4 mm ($T_{4\mathrm{mm}}$) and 10 mm ($T_{10\mathrm{mm}}$) from the electrode surface through the integration of Fourier Analysis SpecTroscopy (FAST) [31], a real-time method for making bioimpedance measurements during electroporation-based treatments. FAST performs electrical impedance spectroscopy (EIS) across a large frequency range in real time (i.e., between H-FIRE bursts) using custom, low-voltage, high-bandwidth rectangular waveforms. One advantage to using FAST over conventional EIS is that the same electronics used to deliver the H-FIRE pulses can also be used for data acquisition for FAST, eliminating the need for a separate set of electronics. In addition to the expenses required with additional equipment, the impedance spectrum acquisition time frame of commercial EIS equipment (∼10 s) is significantly longer than that between therapeutic pulses (∼1 s) employed by electroporation-based therapies. Diagnostic FAST waveforms are interlaced between high voltage therapeutic H-FIRE bursts using a pulse generator. This diagnostic waveform consists of a high-frequency 1-50-1-50 μs (energized time 164 μs) sequence appended to a low-frequency 250-10-250-10 μs (energized time 1 ms) sequence. This concatenated waveform allows for an impedance spectra sweep of 1.8 kHz–4.93 MHz which is critical in the realm of tissue electroporation-based applications. Low-frequency pulses are primarily confined to the extracellular regions. Once pores begin to form in the cellular membranes, more current pathways are formed, lowering the effective impedance until the tissue is fully electroporated. At higher frequencies, currents short the membrane reactance and can penetrate the cell more easily; subsequently, impedance changes at these high frequencies are minimally affected by pore formation. However, in the case of this specific study, no living cells or tissue are used, therefore, considerations of electroporation effects are not necessitated.

The model is based on the development of a state-space model (SSM) that considers tissue as a system with inputs (applied voltage, tissue conductivity, and impedance changes) and outputs (tissue temperature at 4 mm and 10 mm). The state-space model would be easy to implement for estimating temperature from real-time impedance measurements during treatments while keeping all of the crucial aspects of more complicated models. Hence, it may be used for real-time assessment of tissue response or as a theoretical foundation for more sophisticated temperature controls. In this work, the data-driven model was built from experimental results in an agar tissue phantom and then validated using established theoretical models based on numerical modeling.

## 2. Materials and Methods

### 2.1. AGAR Model

**Figure 1:**
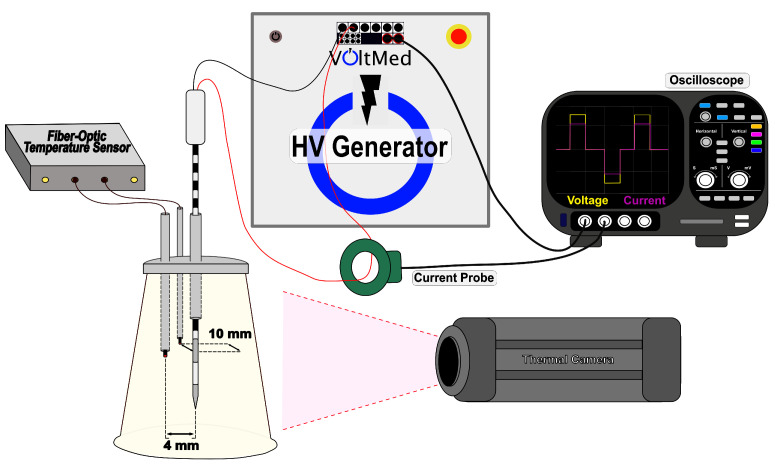
Schematic of the physical experimental setup for recording temperature and impedance measurements during treatment delivered from a high-voltage (HV) generator. A custom 3D printed electrode and fiber-optic temperature holder was fit on top of the agar tissue phantom. Two fiber-optic temperature probes were fed into the canals at a distance of 4 and 10 mm away from the electrode surface. Impedance measurements were calculated from voltage and current measurements collected on the oscilloscope. A thermal camera was placed at a distance 25 cm from the flat edge surface of the tissue phantom.

The experimental design was based on a tissue-mimic agar phantom (Figure 1). Four groups were evaluated to create a comprehensive model: high-voltage high-conductivity (HV-HC), low-voltage low-conductivity (LV-LC), high-voltage low-conductivity (HV-LC), and low-voltage high-conductivity (LV-HC). Data acquisition of n = 5 for each group was conducted (N = 20). A separate agar phantom was created for each sample. Deionized water combined with 1% agar (*w*/*v*) and 0.034% or 0.24% NaCl (*w*/*v*), to achieve 0.1220 or 0.526 S/m conductivities, respectively, were mixed and heated to 90 ${}^{{}^{\circ}}$C. Mixed agar solution was poured into a plastic cup mold with a top diameter of 71 mm, a bottom diameter of 46 mm, and a height of 66 mm and allowed to cool to room temperature. Conductivities of the agar were measured at 37 ${}^{{}^{\circ}}$C to ensure accurate conductive properties of tissues at physiological temperatures. The low conductivity model was set to mimic the conductivity of brain tissue while the high conductivity model was set to mimic initial conductivity of pancreatic tissue at a characteristic frequency of 1.8 kHz [32,33].

### 2.2. Treatment Parameters

Biphasic pulsed electric fields were administered using a custom pulse generator (VoltMed Inc., Blacksburg, Virginia). Treatment voltages were applied at either 1250 V or 2500 V (achieving voltage to distance ratios of 834 and 1666 V/cm, respectively), and delivered at a rate of 1 burst per second for 300 s. A 5-5-5-5 μs waveform with 100 μs of energized time was delivered through a single insertion bipolar probe (diameter = 1.65 mm; electrode exposure = 7 mm; insulation = 8 mm) with voltage and current waveforms monitored with a WaveSurfer 3024 z oscilloscope (Teledyne LeCroy, Chestnut Ridge, NY, USA) equipped with a 1000× high voltage probe (BTX Enhancer 3000, Holliston, MA, USA) and a 10× current probe (3972, Pearson Electronics, Palo Alto, CA, USA).

### 2.3. Real-Time Impedance Acquisition

**Figure 2:**
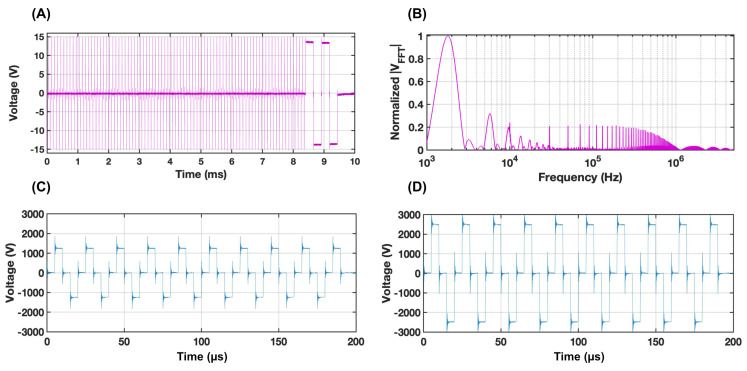
(**A**) The custom diagnostic FAST acquisition waveform delivered between therapeutic voltage bursts creates (**B**) a normalized frequency spectrum of the absolute FFT voltage magnitude, which was then used to collect real-time impedance measurements from our agar phantom. Delivered waveforms for the respective low-voltage (**C**) and high-voltage (**D**) groups are also shown.

FAST impedance measurements were recorded using a 3024z Oscilloscope (Teledyne LeCroy, Chesnut Ridge, NY, USA) and a 1× current probe (2877, Pearson Electronics, Palo Alto, CA, USA). The recorded voltage and current waveforms from the custom FAST waveform (Figure 2A) were converted into the frequency domain (Figure 2B), and the complex tissue impedance was calculated as a ratio of the two following Ohm’s law. For this study, impedance measurements were calculated from peak values at the 1.8 kHz frequency as the agar phantom is unaffected by tissue electroporation effects. The low-frequency impedance decreases as a function of temperature increase. After a considerable number of pulses, the low-frequency impedance mimics the stable high-frequency impedance. Further details on this technique can be found at [31].

### 2.4. Temperature Acquisition

To achieve reproducible conditions between electroporation treatments, a custom-made electrode and temperature sensor holder was developed to precisely position the small fiber-optic temperature sensors (Luxtron m3300; LumaSense, Santa Clara, CA, USA) and single bipolar electrode. The holder was printed on a Form3 printer (FormLabs, USA) with a 25 μm print resolution using FormLabs Clear Resin. The holder consists of a 2.1 mm canal in the center for the electrode, two 1.1 mm canals for the fiber-optic temperature sensors with a center to electrode surface distance of 4 mm and 10 mm, and a depth of 30 mm, placing the temperature probes at a depth parallel with the active top electrode. Prior to treatment, a slice parallel to and at a 10 mm distance from the electrode surface was made to create a flat surface for generation of an immediate post-treatment thermal profile, recorded on a FLIR A325SC thermal camera (FLIR, Wilsonville, OR, USA). Shortly after treatment (within ∼5 s), the agar was sliced along the electrode surface to obtain a thermal profile of the highest temperature areas.

### 2.5. Data Processing

Voltage and current processing and impedance extractions were conducted in MATLAB v.R2022a (MathWorks Inc., Natick, MA, USA). Impedance measurements were transmitted to GraphPad Prism (GraphPad Software, San Diego, CA, USA) for fitting to a one-phase decay line to remove noise, and then sent back to MATLAB for difference calculations. Similarly, collected temperature and impedance measurements were loaded into GraphPad Prism for quadratic line fitting to remove noise and then imported back into MATLAB for calculation of temperature changes. All further data processing and statistical analysis were conducted in GraphPad Prism.

### 2.6. Development of the Mathematical Model

A state-space model was chosen because of its capacity to decompose a complex higher-order differential equation into a sequence of first-order equations that can be easily solved, hence reducing computational burden. State-space control utilizes differential equations describing the time domain of the system using state variables in vector form. This makes it easy to evaluate the system using simple matrix algebra, which also enables the evaluation of complex multi-input multi-output (MIMO) systems. Other control system approaches require complicated Laplace transforms and Fourier transforms to transfer the system’s time domain representation—supplied as a complex set of differential equations—into the frequency domain—given as a collection of algebraic equations.

**Figure 3:**
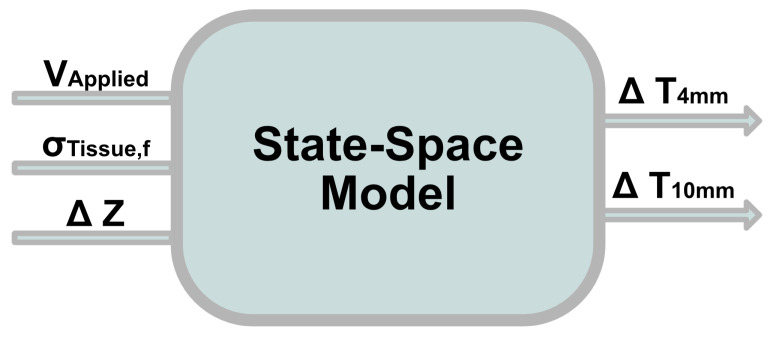
Schematic proposing the model inputs of change in impedance (ΔZ), applied voltage (V), and initial tissue conductivity under the influence of a specific frequency waveform ($\sigma_{Tissue,f}$) to be fed into a fit state-space model to give real-time estimation of the temperature rise at distances 4 mm and 10 mm away from the electrode surface.

The agar tissue mimic was seen as a dynamic system with the applied voltage (V), tissue identification by electrical conductivity (S) at the applied characteristic frequency, and intra-treatment impedance change measurements (ΔZ) as the input signals, and the temperatures measured at depths of 4 mm ($T_{4}$) and 10 mm ($T_{10}$) as the output signals (Figure 3). State-space modeling only requires unique state equations for values influencing the system that cannot be deduced from other model states already included; thus, a separate state for applied waveform is not necessary as its influence can be deduced from the tissue conductivity.

We consider a system governed by the following continuous-time identified state-space model:

(1) $$\dot{x}=Ax\left(t\right)+Bu\left(t\right)+Ke\left(t\right)$$

(2) x(0)=0

(3) y=Cx(t)+Du(t)+e(t)

where *x* is the state vector, *u* is the input vector, *y* is the output vector, and *K* is the disturbance component. *A*, *B*, *C* and *D* are the state-space matrices which describe the system dynamics. By default, *D* is set to 0 for dynamic systems, indicating that the system has no feedthrough.

The state-space model was estimated by utilizing the numerical algorithms for subspace state-space identification (N4SID). Implementation of this method in MATLAB’s System Identification toolbox uses input–output data to construct the model’s weighting matrices. This method can be viewed as a type of multi-step, constrained prediction error method utilizing linear regression to solve the system matrix. The algorithm was configured to compute a fourth order SSM. Further details on the methodology for the N4SID method are outlined in Appendix A.

### 2.7. Validation of the Mathematical Model against FEM

After developing an SSM based on results from experimentally collected data, a finite element model was developed to create new test data to assess the validity of the state-space model. A three-dimensional Comsol Multiphysics 6.0 (Comsol, Stockholm, Sweden) model was designed to replicate the experimental conditions of the agar phantom.

The distribution of electrical fields and thermal effects were computed using standard methods [34,35]. The electric potential distribution at the end of a given pulse was calculated with a modified Laplace equation under the electroquasistatic approximation (Equation (4)), and the resulting electric field distribution was calculated with Equation (5).

(4) −∇·(σ(T)∇Φ)=0

(5) $$\vec{E}=-\nabla \Phi$$

where σ is the electrical conductivity, $\vec{E}$ is the local field magnitude, *T* is the temperature, and Φ is the local electric potential.

Following the computation of the electric field distribution, tissue temperature was computed using the general heat conduction equation with the addition of a Joule heating term ($Q_{J}$),

(6) $$\rho c_{p}\frac{\partial T}{\partial t}=\nabla \cdot \left(k\nabla T\right)+Q_{J}$$

(7) $$Q_{J}=\sigma {\left|-\nabla \phi \right|}^{2}\cdot \frac{p}{\tau }$$

where ρ is the density of the medium; $c_{p}$ is the specific heat; *k* is the thermal conductivity; *p* is the burst on-time ($100\times 10^{-6}s$) and τ is the period of the burst delivery (1 s). The initial conditions were set to match those of the experimental conditions with the outer boundaries of the geometry being assigned a convective heat flux due to air convective cooling with an external temperature of $T_{ext}$ = 18.8 ${}^{{}^{\circ}}$C. Due to the discrepancies between agar conductivity being measured at physiological temperatures (37 ${}^{{}^{\circ}}$C), set conductivities ($\sigma_{0})$ were scaled by the temperature of the agar at the time of pulsing ($T_{agar}$) to match the conductivity of the agar at the time of pulsing ($\sigma_{f})$ by using the following:

(8) $$\sigma_{f}\left(T\right)=\sigma_{0}*(1+\alpha *\left(T_{agar}-37^{\;{}^{\circ}}C\right)$$

producing initial conductivities of 0.331 S/m, 0.085 S/m, 0.093 S/m, and 0.331 S/m for the HV-HC, LV-LC, HV-LC and LV-HC groups, respectively. Material properties of the agar phantom were set to those of water. All other thermal and electrical conditions may be found in Table 1.

## 3. Results

### 3.1. Experimental Data Results

**Figure 4:**
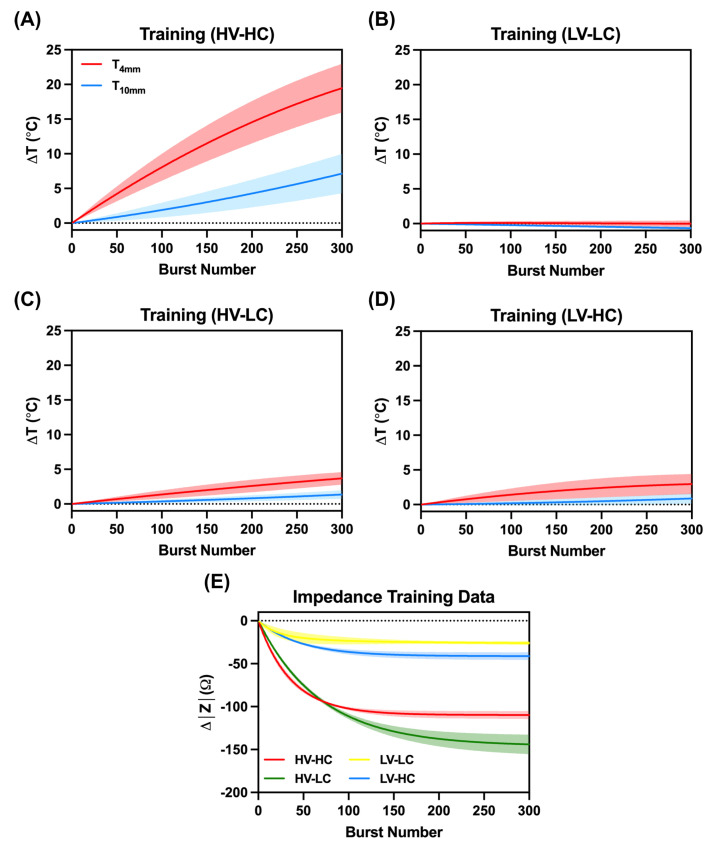
Results of the experimental temperature changes for the (**A**) High Voltage-High Conductivity (HV-HC), (**B**) Low Voltage-Low Conductivity (LV-LC), (**C**) High Voltage-Low Conductivity (HV-LC), and (**D**) Low Voltage-High Conductivity (LV-HC) groups as well as the (**E**) recorded changes in impedance measurements from the agar tissue phantom. Shaded regions relate to standard deviations within each group.

The recorded temperature and impedance change data points were used as training data along with known parameters such as voltage and tissue conductivity (Figure 4).

### 3.2. Model Validation with Training and Validation Data Sets

Selection of the final model was chosen through analysis of the accuracy of the training data being tested through the model, as well as a validation set in which specific data sets from each group were omitted from training the model.

**Figure 5:**
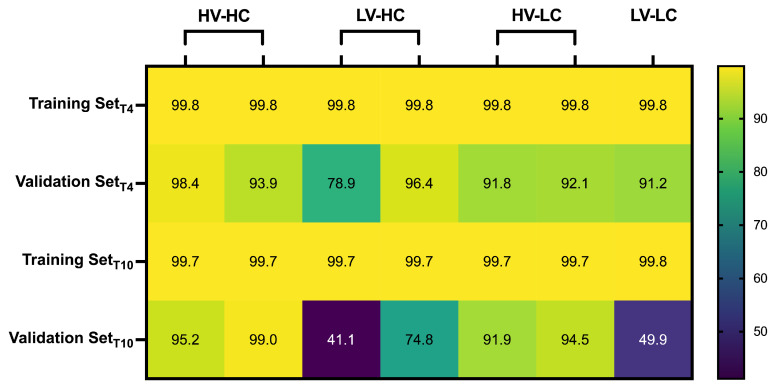
A heat map distribution of the percent accuracies for the respective High Voltage-High Conductivity (HV-HC), Low Voltage-High Conductivity (LV-HC), High Voltage-Low Conductivity (HV-LC), and Low Voltage-Low Conductivity (LV-LC) training and validation sets during the training phase of the model formation.

The training and validation data set accuracies are shown in the heat map tiles (Figure 5) for $T_{4}$ and $T_{10}$. Specification of the grouping where a data set was omitted for use as the validation data set is specified by the column titles. The fit accuracy was calculated by:

(9) $$fit=100*(1-\frac{|y_{pred}-y|}{\left|y-mean(y)\right|})$$

where *y* is the validation data point value and $y_{pred}$ is the predicted model from the output.

### 3.3. Model Implementation with Test Data

We validated our data-driven state-space model against a COMSOL finite element model, replicating our experimental setup. The calculated outputs of the COMSOL model were impedance and temperature at 4mm and 10mm for each voltage and conductivity condition. By importing the extracted impedance into MATLAB, we were then able to obtain predicted temperatures with our state-space model, trained on experimental data. These predicted temperatures were then used to calculate fit accuracy and maximum absolute error (Table 2).

**Figure 6:**
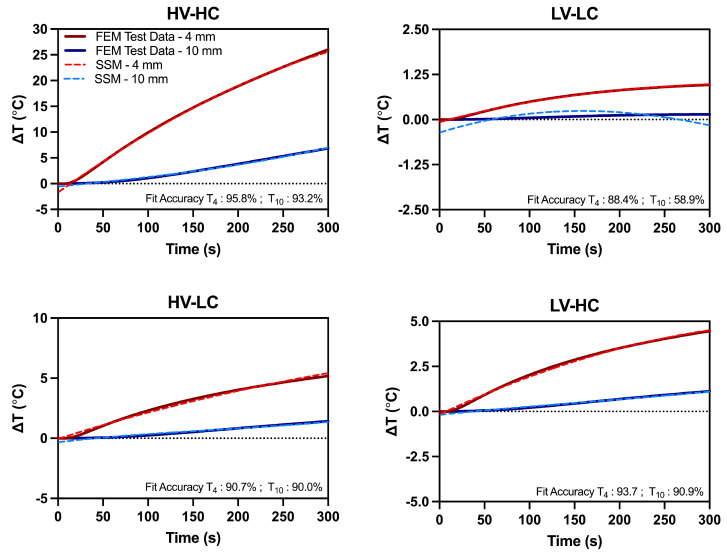
Impedance change measurements extracted from the FEM were used as test data fed through the resulting state-space model. Temperature rise from the FEM and the resulting outputs from the state-space model for the High Voltage-High Conductivity (HV-HC), Low Voltage-Low Conductivity (LV-LC), High Voltage-Low Conductivity(HV-LC), and Low Voltage-Low Conductivity (LV-LC) groups are plotted. Note: *y*-axis is scaled to view the largest absolute errors in model performance.

We found that the mathematical model provided excellent estimations of temperature rise, with above 90% fit accuracy for all conditions, except for the LV-LC condition. The discrepancy for this point can be attributed to the low absolute change in temperature. At the 10 mm distance, the temperature increase is negligible, and the model was not able to fit such a small change. Though the LV-LC had the lowest fit accuracy, the absolute error in temperature prediction was under 0.5 ${}^{{}^{\circ}}$C. Further, all the test conditions had a maximum absolute error of under 0.5 ${}^{{}^{\circ}}$C, except for the HV-HC, which has a maximum absolute error of 1.45 and 0.52 ${}^{{}^{\circ}}$C for the 4 mm and 10 mm positions, respectively. The state-space model temperature predictions and finite element temperature simulations at both distances are given in Figure 6 for each voltage and conductivity condition.

The overall model quality was found to be 9.116e-10 as calculated by Akaike’s Final Prediction Error (FPE):

(10) $$FPE=V(1+\frac{2d}{N})$$

where *d* is the number of estimated parameters and the loss function, *V*, is represented by:

(11) $$V=det(\frac{1}{N}\sum_{1}^{N}\epsilon \left(t,\theta_{N}\right){\left(\epsilon \left(t,\theta_{N}\right)\right)}^{T})$$

where *N* represents the number of values in the estimation data set, ϵ(t) is the respective prediction error, and ${\left(\theta \right)}_{N}$ is the estimated parameters.

## 4. Discussion

**Figure 7:**
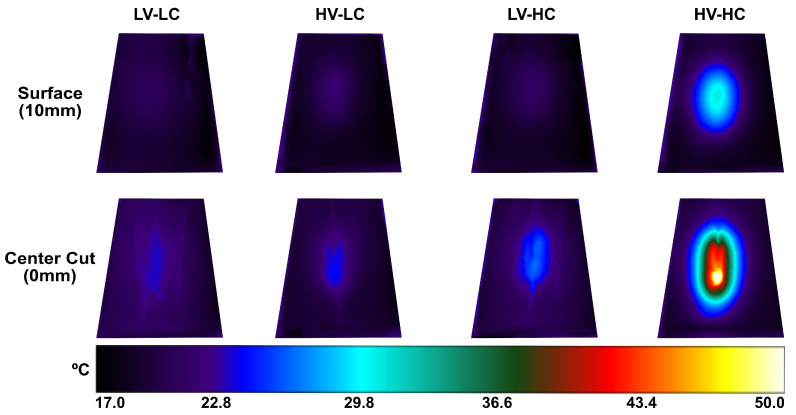
Snapshots taken from the FLIR thermal camera immediately after pulsing at the phantom surface (a distance 10 mm away from the electrode), and shortly after pulsing (within ∼5 s) at the center of the phantom in the plane of the electrode surface.

The goal of this study was to evaluate the feasibility of real-time temperature monitoring during an IRE or H-FIRE procedure without external temperature probes or devices. This feasibility study demonstrates a proof-of-concept construction of a black-box model to forecast tissue temperature rise during the ablation process based on observations of real-time impedance changes. In addition to not requiring additional sensors, one of the greatest potential advantages to implementing a state-space model is its ability to provide spatial data at several locations which would otherwise be challenging, if not impossible, in vivo. The model yielded a reasonably good estimate of temperature rise in most cases; however, in certain instances, considerable inaccuracies were detected (e.g., those in which the total treatment temperature rise was on average <1.5 ${}^{{}^{\circ}}$C). Despite these discrepancies at low temperatures, temperature rises of this magnitude are not relevant in the context of clinical temperature monitoring. From Figure 7, it is evident that the conductivity of the agar plays the greatest role in temperature fluctuations and thus suggests that thermal monitoring may only be necessary in the treatment of tissues with high conductivities (e.g., pancreas, prostate, etc.).

Due to the fact that the electrical and thermal properties of the gel may differ from those of normal tissue—for instance, the gel was cooler and was not perfused—the absence of our model accounting for flow-rate perfusion, which in the clinical setting should permit a considerable degree of variability, means that these temperature curves cannot be directly applied to the clinical environment. Even though we have focused on the development of wholly data-driven prediction models, future computational modeling could be used to incorporate more biophysical information in the model’s input parameters as a means for expanding the utility of the model without making it entirely data-driven. This strategy could improve the accuracy of predictions, as we believe the proposed technique could be enhanced by combining new data from a range of electrical and thermal tissue properties. Moreover, state-space models built on computationally driven data could make it simpler to create multi-output models with more locations for temperature predictions. Future studies may also consider developing a model which considers a multi-electrode setup. However, such geometries would introduce additional variables to be fit including electrode spacing and exposure.

In addition, the process could be more complicated in the tissue domain due to the complex impedance spectrum in living tissue [38,39,40,41,42]. Because low-frequency impedance measurements are primarily confined to the extracellular region prior to tissue electroporation, membrane permeabilization during treatment has a substantial effect on impedance alterations. However, high-frequency impedance measurements, which correspond to currents that short the membrane reactance and penetrate the cell membrane, are less vulnerable to membrane pore formation. Thus, in the case of a tissue model, high-frequency impedance measurements may be used as a benchmark to identify impedance changes caused primarily by thermal effects.

## 5. Conclusions

State-space models can be created to predict tissue temperature increases during H-FIRE treatment by utilizing impedance changes calculated from rapid impedance spectroscopy, applied voltage, and the conductivity of the tissue as described at the characteristic frequency of the applied waveform. The best model produces a reasonably accurate prediction of tissue temperature with an overall model output to test data fit of 95.8% and 93.22% for $T_{4}$ and $T_{10}$, respectively. The largest model discrepancies are observed in the scenario where the least amount of temperature rise is observed overall (temperature increases <1.5 ${}^{{}^{\circ}}$C). Test data validation of the model suggests that the model is successful at reasonably predicting temperature increases in cases where temperature increases are of clinical relevance.

## Figures and Tables

**Table 1:** Electrical and Thermal Properties for Computational Modeling.

Material	Parameter	Value	Units	Ref
Agar	Density, ρ Specific heat, c_p_ Thermal conductivity, *k* Temperature coefficient, α	998 4182 0.598 2	kg/m^3^ J/(kg·K) W/(m·K) %/${}^{{}^{\circ}}$C	[36] [36] [36] [36]
Insulation	Density, ρ Specific heat, c_p_ Thermal conductivity, *k* Electrical conductivity, σ	2329 700 0.2 1 × 10^−12^	kg/m^3^ J/(kg·K) W/(m·K) S/m	[26] [26] [37] [26]
Stainless Steel	Density, ρ Specific heat, c_p_ Thermal conductivity, *k* Electrical conductivity, σ	7900 500 15 2.22 × 10^6^	kg/m^3^ J/(kg·K) W/(m·K) S/m	[26] [26] [26] [26]

**Table 2:** Quantitative metrics on the validity of the model following feedthrough of FEM test data.

	Temperature Distance	HV-HC	LV-LC	HV-LC	LV-HC
**Fit Accuracy (%)**	T${}_{4\mathrm{mm}}$	95.8	88.4	90.7	93.7
	T${}_{10\mathrm{mm}}$	93.2	58.9	90.0	90.9
**Maximum Absolute Error (${}^{{}^{\circ}}$C)**	T${}_{4\mathrm{mm}}$	1.45	0.06	0.30	0.19
	T${}_{10\mathrm{mm}}$	0.52	0.35	0.31	0.17

## Data Availability

The data presented in this study are available upon request from the corresponding author.

## Abbreviations

The following abbreviations are used in this manuscript:

FAST	Fourier Analysis SpecTroscopy
FEM	Finite Element Modeling
H-FIRE	High-Frequency Irreversible Electroporation
IRE	Irreversible Electroporation
N4SID	Numerical Algorithms for Subspace State-Space Identification
PEF	Pulsed Electric Field
SSM	State-Space Model

## Appendix A

### N4sid Oblique Projection by LQ Decomposition

The N4SID subspace method uses LQ decomposition, singular value decomposition, and applies an oblique projection approach for input and output Hankel blocks to produce subspace weighting matrices [43,44]. Past and future block Hankel matrices for inputs $U_{p},U_{f}$ and outputs $Y_{p},Y_{f}$ are constructed from experimental input and output data,

$$U_{p}=\left[\begin{array}{cccc} u_{0} & u_{1} & \ldots & u_{N-1}\\ u_{1} & u_{2} & \ldots & u_{N}\\ \vdots & \vdots & \ddots & \vdots \\ u_{2k-1} & u_{2k} & \ldots & u_{2k+N-2} \end{array}\right]U_{f}=\left[\begin{array}{cccc} u_{k} & u_{k+1} & \ldots & u_{k+N-1}\\ u_{k+1} & u_{k+2} & \ldots & u_{k+N}\\ \vdots & \vdots & \ddots & \vdots \\ u_{k-1} & u_{k} & \ldots & u_{k+N-2} \end{array}\right]$$

$$Y_{p}=\left[\begin{array}{cccc} y_{0} & y_{1} & \ldots & y_{N-1}\\ y_{1} & y_{2} & \ldots & y_{N}\\ \vdots & \vdots & \ddots & \vdots \\ y_{2k-1} & y_{2k} & \ldots & y_{2k+N-2} \end{array}\right]Y_{f}=\left[\begin{array}{cccc} y_{k} & y_{k+1} & \ldots & y_{k+N-1}\\ y_{k+1} & y_{k+2} & \ldots & y_{k+N}\\ \vdots & \vdots & \ddots & \vdots \\ y_{k-1} & y_{k} & \ldots & y_{k+N-2} \end{array}\right]$$

in which N gives us the dimensions of the matrix size and k gives us the number of elements in each matrix.

Our output state-space equation can be defined as

(A1) y(t)=Cx(t)+Du(t)

The N4SID subspace method determines that our output matrix, $Y_{k}\left(t\right)$, can thus be found through the following relationship:

$$\underset{Y_{k}\left(t\right)}{\underset{︸}{\left[\begin{array}{c} y(t)\\ y(t+1)\\ \vdots \\ y(t+k-1) \end{array}\right]}}=\underset{O_{k}}{\underset{︸}{\left[\begin{array}{c} C\\ CA\\ \vdots \\ CA^{k-1} \end{array}\right]}}\underset{X_{0}}{\underset{︸}{x(t)}}+\underset{\Psi_{k}}{\underset{︸}{\left[\begin{array}{cccc} D & 0 & \ldots & 0\\ CB & D & \ldots & \vdots \\ \vdots & & & 0\\ CA^{k-2}B & \ldots CB & \ldots & D \end{array}\right]}}\underset{U_{k}\left(t\right)}{\underset{︸}{\left[\begin{array}{c} u(t)\\ u(t+1)\\ \vdots \\ u(t+k-1) \end{array}\right]}}$$

with $O_{k}$ representing the observability matrix, $X_{0}$ representing the state initial state, $\Psi_{k}$ representing the Toeplitz matrix designating the shift-invariant properties of the system, and $U_{k}$ is the input matrix. Thus, the output matrix can be represented by

(A2) $$Y_{f}=O_{k}X_{f}+\Psi_{k}U_{f}.$$

In order to solve for the solution to our subspace weighting factors [A B C D], we must perform LQ decomposition of the data matrix ($W_{p}$) into the product of a lower triangular matrix (L) and a unitary matrix (Q):

(A3) $$W_{p}=\left[\begin{array}{c} U_{p}\\ Y_{p} \end{array}\right]$$

(A4) $$\left[\begin{array}{c} U_{f}\\ W_{p}\\ Y_{f} \end{array}\right]=\left[\begin{array}{ccc} R_{11} & 0 & 0\\ R_{21} & R_{22} & 0\\ R_{31} & R_{32} & 0 \end{array}\right]\left[\begin{array}{c} Q_{1}^{T}\\ Q_{2}^{T}\\ Q_{3}^{T} \end{array}\right]$$

The resulting equations are as follows:

(A5) $$U_{f}=R_{11}Q_{1}T$$

(A6) $$W_{p}=R_{21}Q_{1}^{T}+R_{22}Q_{2}^{T}$$

(A7) $$Y_{f}=R_{31}Q_{1}^{T}+R_{32}Q_{2}^{T}$$

Through substitution of terms, we end up with the following relationship:

(A8) $$Y_{f}=\left(R_{31}-R_{32}R_{22}^{\#}R_{21}\right)R_{11}^{-1}U_{f}+R_{32}R_{22}^{\#}W_{p}$$

suggesting that the two terms $U_{f}$ and $W_{p}$ exist in two different sub-spaces with no overlapping bases. Thus from this realization, we can conclude that from Equation (A2),

(A9) $$O_{k}X_{f}=R_{32}R_{22}^{\#}W_{p}$$

and use singular value decomposition of $R_{32}R_{22}^{\#}W_{p}$ to solve for $O_{k}$ and $X_{f}$.

(A10) $$R_{32}R_{22}^{\#}W_{p}=\left[\begin{array}{cc} U_{1} & U_{2} \end{array}\right]\left[\begin{array}{cc} \Sigma_{1} & 0\\ 0 & 0 \end{array}\right]\left[\begin{array}{c} V_{1}^{T}\\ V_{2}^{T} \end{array}\right]=U_{1}\Sigma_{1}V_{1}^{T}$$

The output of Equation (A10) can therefore be split between $O_{k}$ and $X_{f}$:

(A11) $$O_{k}=U_{1}\Sigma_{1}^{1/2}T$$

(A12) $$X_{f}=T^{-1}\Sigma_{1}^{1/2}V_{1}^{T}$$

By further decomposing $X_{f}$, we can define the series of states:

(A13) x(k),x(k+1),…x(k+N−1)

By combining the states and input–output data, we end up with the following matrices:

(A14) $${\bar{X}}_{k}=\left(x\left(k\right)\ldots x\left(k+N-2\right)\right)$$

(A15) $${\bar{X}}_{k+1}=\left(x\left(k+1\right)\ldots x\left(k+N-1\right)\right)$$

(A16) $${\bar{U}}_{k|k}=\left(u\left(k\right)\ldots u\left(k+N-2\right)\right)$$

(A17) $${\bar{Y}}_{k|k}=\left(y\left(k\right)\ldots y\left(k+N-2\right)\right)$$

and presented in the following state and measurement equations:

(A18) $$\left[\begin{array}{c} {\bar{X}}_{k+1}\\ {\bar{Y}}_{k|k} \end{array}\right]=\left[\begin{array}{cc} A & B\\ C & D \end{array}\right]\left[\begin{array}{c} {\bar{X}}_{k}\\ {\bar{U}}_{k|k} \end{array}\right]$$

through which our weighting matrices [A B C D] can be solved through a least squares regression:

(A19) $$\left[\begin{array}{cc} \hat{A} & \hat{B}\\ \hat{C} & \hat{D} \end{array}\right]=\left[\begin{array}{c} {\bar{X}}_{k+1}\\ {\bar{Y}}_{k|k} \end{array}\right]{\left[\begin{array}{c} {\bar{X}}_{k}\\ {\bar{U}}_{k|k} \end{array}\right]}^{T}{\left[\begin{array}{c} \left[\begin{array}{c} {\bar{X}}_{k}\\ {\bar{U}}_{k|k} \end{array}\right]{\left[\begin{array}{c} {\bar{X}}_{k}\\ {\bar{U}}_{k|k} \end{array}\right]}^{T} \end{array}\right]}^{-1}$$

For our specific system, the following weighting matrices were produced:

A= $\left[\begin{array}{ccccc} & x1 & x2 & x3 & x4\\ x1 & 0.002906 & -0.0004279 & -0.001115 & 0.001509\\ x2 & 70.003136 & 0.003182 & 0.008007 & 0.0006536\\ x3 & 0.002613 & -0.005233 & -0.006247 & -0.0004292\\ x4 & -0.002367 & -0.00598 & -0.001739 & -0.00521 \end{array}\right]$

B= $\left[\begin{array}{cccc} & dZ & V & S\\ x1 & 2.941e-07 & 4.348e-08 & 8.484e-06\\ x2 & -1.581e-07 & -3.313e-08 & -5.155e-05\\ x3 & 5.258e-07 & 3.26e-08 & 3.914e-05\\ x4 & -2.326e-06 & -1.432e-07 & 8.647e-06 \end{array}\right]$

C= $\left[\begin{array}{ccccc} & x1 & x2 & x3 & x4\\ T4mm & 238.5 & -74.14 & -0.6752 & 0.2305\\ T10mm & 98.88 & 6.118 & -0.1381 & 0.1084 \end{array}\right]$

D= $\left[\begin{array}{cccc} & dZ & V & S\\ T4mm & 0 & 0 & 0\\ T10mm & 0 & 0 & 0 \end{array}\right]$

K= $\left[\begin{array}{ccc} & T4mm & T10mm\\ x1 & -6.139e-05 & 0.002894\\ x2 & -0.003739 & 0.009211\\ x3 & -0.1299 & 0.2822\\ x4 & -0.1849 & 0.7076 \end{array}\right]$
